# Hub circRNAs in cancer: dual regulation of metastasis and immune evasion

**DOI:** 10.3389/fimmu.2026.1885182

**Published:** 2026-09-18

**Authors:** Junyu Jia, Zhaokun Liu, Yujie Zhao, Chang Gao, Tongyue Yang, Zhen Ye, Junni Liu

**Affiliations:** 1 Department of Geriatrics, Shandong Provincial Hospital Affiliated to Shandong First Medical University, Jinan, Shandong, China; 2 School of Ophthalmology, Shandong First Medical University, Jinan, Shandong, China; 3 College of Life Sciences, Shandong Normal University, Jinan, Shandong, China; 4 School of Biological Sciences, University of Aberdeen, Aberdeen, United Kingdom; 5 School of Clinical and Basic Medical Sciences, Shandong First Medical University, Jinan, China

**Keywords:** EMT, hub circRNAs, immune evasion, tumor metastasis, tumor microenvironment

## Abstract

Circular RNAs (circRNAs) are a class of highly stable non-coding RNAs that play pivotal roles in cancer progression by concurrently modulating tumor metastasis and immune evasion. Among them, a subset termed hub circRNAs coordinates these two processes within the same tumor context through multiple regulatory mechanisms. Hub circRNAs facilitate tumor cell migration, invasion, and colonization at distant sites, while simultaneously impairing the function of immune effector cells and remodeling the tumor microenvironment into an immunosuppressive state. These dual effects can be propagated to distal microenvironments via extracellular vesicles, thereby amplifying both metastasis and immunosuppression. To organize the available evidence, we propose a provisional three-tiered stringent/convergent/network-informed (S/C/N) evidence-mapping framework that integrates evidence across studies and organizes the type and completeness of functional, mechanistic, and network-informed support. Finally, we highlight current research gaps, outline the potential of hub circRNAs as biomarkers and therapeutic targets, and discuss experimental and methodological considerations for pan-cancer investigations. This review offers a functional perspective for understanding hub circRNAs as regulators linking metastasis with immune evasion and guides future efforts to investigate their dual functions in translational settings.

## 1 Introduction

Cancer initiation and progression involve intricate dysregulation of gene expression networks, with abnormalities in RNA processing emerging as a key research focus (1, 2). Disruptions in processes such as alternative splicing and pre-mRNA stability can lead to abnormal expression of oncogenic or tumor-suppressor transcripts, promoting malignant tumor development (3, 4).

CircRNAs are a class of non-coding RNAs produced from pre-mRNA via back-splicing. Their covalently closed structure provides higher stability than linear RNAs (5, 6). CircRNA biogenesis is tightly regulated by intronic complementary sequences and RNA-binding proteins (RBPs) (7). Advances in high-throughput sequencing have enabled the identification of thousands of circRNAs in both normal and tumor tissues, many of which show disease relevance (8).

CircRNAs participate in cancer progression through multiple regulatory modes, including miRNA sponging, circRNA–RBP interactions, protein scaffolding or decoy functions, encoded peptides, and extracellular vesicle-mediated intercellular communication (9, 10). Through these mechanisms, circRNAs can influence not only tumor cell proliferation and survival, but also malignant phenotypes directly related to disease dissemination and therapeutic response.

During metastatic progression, circRNAs regulate EMT, tumor cell migration and invasion, angiogenesis, vascular permeability, and metastatic niche formation (11–14). During immune evasion, circRNAs can modulate immune checkpoint expression, impair immune effector cell activity, and reshape the tumor microenvironment into an immunosuppressive state, thereby influencing antitumor immunity and immunotherapy response (15, 16). These properties also give circRNAs potential translational value as stable biomarkers and potential therapeutic targets in cancer research (8, 11, 17).

However, most existing studies still discuss circRNA-mediated metastasis and circRNA-mediated immune evasion as separate topics. This separation may underestimate circRNAs that function at the intersection of metastatic and immune-regulatory programs. In reality, EMT programs, PD-L1 signaling, TGF-β/STAT3 activation, macrophage polarization, metabolic reprogramming, and exosome-mediated communication can contribute to both metastatic progression and immune evasion (11, 17). Therefore, a cross-phenotype perspective is needed to identify circRNAs that may exert regulatory influence across these two malignant programs.

To address this gap, this review introduces the concept of hub circRNAs and proposes a provisional stringent/convergent/network-informed (S/C/N) evidence-mapping framework to organize existing findings according to the type and completeness of functional, mechanistic, and network-informed evidence. In the following sections, we first provide detailed definitions, evidence-assignment principles, and representative examples of this framework and then further discuss its current limitations, potential clinical implications, and future validation directions.

## 2 CircRNA biogenesis, characteristics, and core functions

### 2.1 Mechanisms of CircRNA biogenesis

The vast majority of circRNAs arise from back-splicing of pre-mRNA, in which a downstream 5′ splice donor is covalently joined to an upstream 3′ splice acceptor, forming a circRNA molecule (18). Based on sequence composition, circRNAs are mainly classified as exonic circRNAs (ecircRNA), exon-intron circRNAs (EIciRNA), and intronic circRNAs (ciRNA) (19).

Three primary models have been proposed for back-splicing. In the intron-pairing-driven model, complementary intronic sequences directly bring splice sites into proximity, facilitating back-splicing. The RBP-mediated circularization model posits that specific RBPs act as molecular bridges, juxtaposing splice sites to promote back-splicing. In the lariat-driven model, lariat structures formed after exon skipping undergo internal splicing, producing circRNAs (18, 20).

EcircRNAs and EIciRNAs are predominantly generated through the back-splicing mechanisms described above. EIciRNAs, retaining partial introns, are localized in the nucleus and can modulate host gene transcription (21, 22). Intronic circRNAs primarily arise from intron lariats that escape debranching degradation (23). CircRNAs encoded by the mitochondrial genome are produced independently of the nuclear spliceosome, and their biogenesis mechanisms remain under investigation (24).

### 2.2 Core characteristics of CircRNAs

Unlike linear RNAs, circRNAs lack a 5′ cap and a poly(A) tail, adopting a covalently closed circular conformation. This closed-loop conformation renders circRNAs resistant to exonuclease-mediated degradation, conferring exceptional stability and a prolonged half-life, enabling their accumulation in slowly dividing cells (25, 26). CircRNA expression is highly tissue-, cell-, and developmental stage-specific, with some circRNAs even more abundant than their linear counterparts (27). In terms of subcellular localization, ecircRNAs are predominantly cytoplasmic, whereas EIciRNAs and ciRNAs are primarily nuclear (28). Their nucleocytoplasmic transport is selectively mediated by DDX39A and DDX39B in a length-dependent manner (29). Moreover, many functionally important circRNAs (e.g., ciRS-7) are highly conserved across species, indicating they are subject to evolutionary selection (30).

### 2.3 Main functional mechanisms of CircRNAs

Acting as miRNA sponges and engaging in competing endogenous RNA (ceRNA) network regulation constitutes the canonical functional mechanism of circRNAs. For example, ciRS-7 harbors more than 70 binding sites for miR-7, effectively sequestering and inhibiting its activity, thus alleviating the repression of downstream target genes and precisely modulating the post-transcriptional gene expression network (27, 31).

CircRNAs also serve as platforms for protein interactions, with regulatory modes that include protein sponges, molecular decoys, scaffolds, and recruiters. For example, circAKT3 acts as a molecular scaffold, simultaneously binding ribosomal proteins RPS27A and RPL11, enhancing their interaction and thereby promoting tumor progression (32, 33).

Some circRNAs possess translation capabilities independent of the 5′ cap structure, initiating translation via internal ribosome entry sites (IRES) or N6-methyladenosine (m^6^A) modification. For example, circTRIM1 utilizes an IRES element to translate a functional protein, TRIM1-269aa, challenging the traditional view of circRNAs as purely non-coding molecules and adding a new dimension to their functional diversity (25, 34). The biogenesis and functional mechanisms of circRNAs are summarized in **Figure 1**.

**Figure 1:**
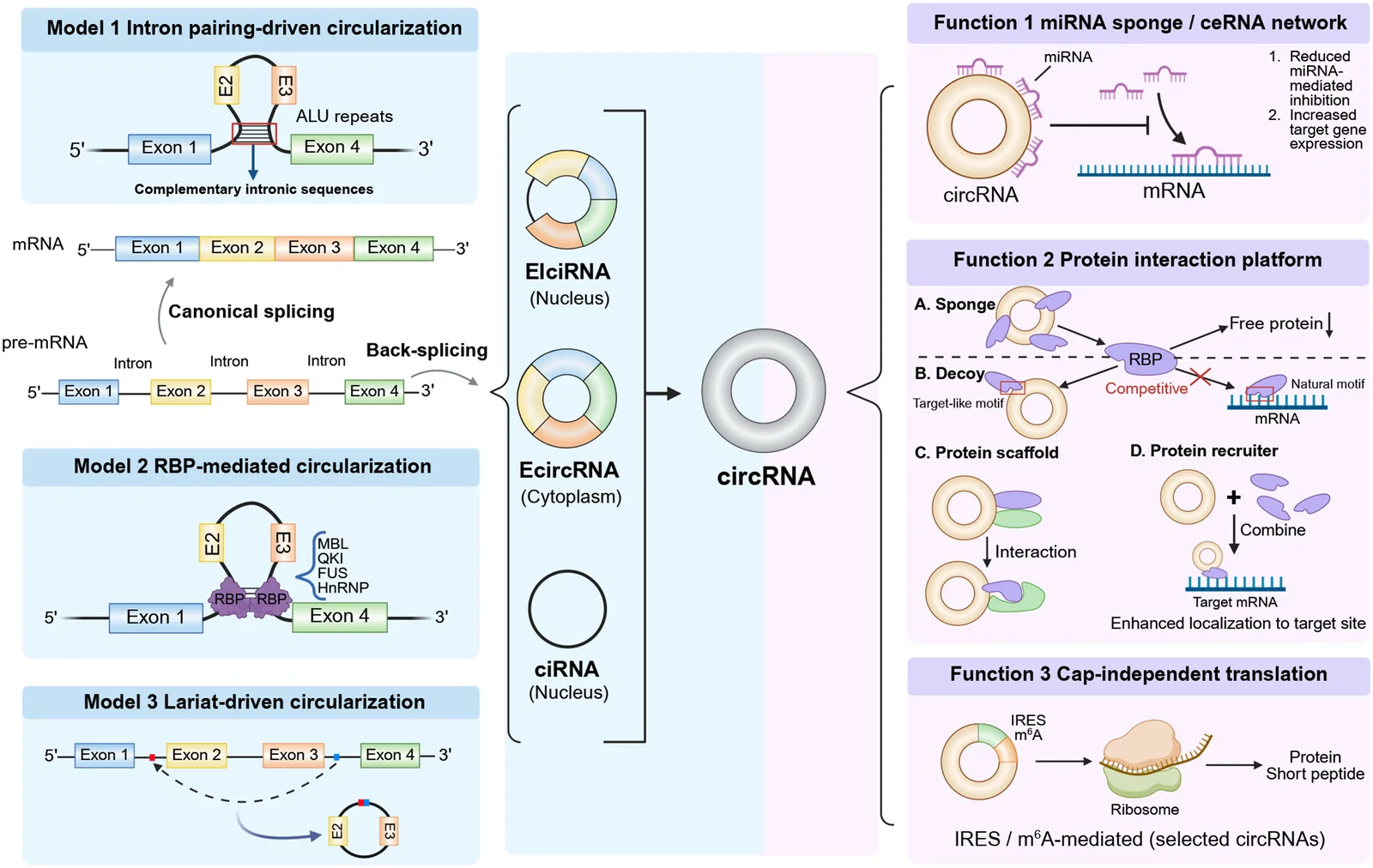
Biogenesis and main functions of circRNAs. CircRNAs are mainly produced from pre-mRNA by back-splicing, which can be driven by intron pairing, RNA-binding proteins, or lariat formation. Different circRNA types show distinct localization patterns, and they may function as miRNA sponges, protein interaction platforms, or, in some cases, templates for IRES- or m^6^A-mediated translation. Created in BioRender. Zhao, Y (2026). https://BioRender.com/4i3z8p6.

## 3 Molecular mechanisms of CircRNA-driven tumor metastasis

Tumor metastasis follows the invasion–metastasis cascade: local invasion (accompanied by EMT), intravasation, survival in the circulation, arrest at distant organ sites, extravasation, micrometastasis formation, and metastatic colonization (35). During this cascade, circRNAs promote tumor metastasis through multilayered mechanisms, including ceRNA networks, RBP interactions, reprogramming of signaling pathways, and intercellular communication via extracellular vesicles (31, 36).

### 3.1 CircRNAs as ceRNAs regulating metastasis: from miRNA de-repression to EMT activation

The ceRNA mechanism is the classical paradigm for circRNA-mediated metastasis regulation. Among these, ciRS-7 (also known as CDR1as) is one of the best-characterized examples of this mechanism and harbors over 70 binding sites for miR-7, making it one of the densest natural miRNA sponges identified to date. ciRS-7 has been confirmed to be closely associated with more than ten cancer types (37, 38). miR-7 is a well-established tumor-suppressive miRNA, targeting key oncogenic drivers of proliferation and invasion, including EGFR, Raf1, and PI3K. By extensively sponging miR-7, ciRS-7 relieves translational inhibition of these target genes, consequently activating the downstream MEK/ERK and PI3K/AKT signaling pathways (39, 40). This signaling activation further upregulates core EMT transcription factors, such as Snail and Zeb1, driving E-cadherin loss and mesenchymal phenotype acquisition and thereby promoting tumor cell migration, invasion, and metastatic colonization (39, 41). In recent years, this ceRNA network has expanded further: ciRS-7 can sponge miR-1299 to target the EGFR signaling pathway and inhibit autophagy in esophageal squamous cell carcinoma (42); it can also act as a ceRNA for RAF-1/PIK3CD to activate the MAPK/AKT pathway, promoting oral squamous cell carcinoma metastasis (43); additionally, ciRS-7 can bind IGF2BP1 to stabilize SLUG mRNA, enhancing cervical cancer metastasis (44). The paradigmatic significance of ciRS-7 lies in its clear illustration of the stepwise amplification cascade model—“single circRNA → ceRNA network perturbation → signaling pathway → EMT transcriptional program”—providing a core framework for understanding how ceRNA mechanisms convert molecular interactions into metastatic phenotypes. The ceRNA-based regulation of EMT-related programs has also been reported in non-tumor epithelial contexts, as exemplified by circHIPK3, while circPVT1 provides a cancer-specific example (45, 46). **Supplementary Table 1** provides a multi-column overview of circRNA–miRNA interactions across tumor types, summarizing targets and pathway effects.

### 3.2 CircRNA–RBP interactions: extending to protein-level regulation

Direct interactions between circRNAs and RBPs transcend RNA-level competition, reshaping the metastasis regulatory network at two levels: protein abundance and complex assembly (47). At the protein stability level, circPRKD3 exemplifies a pro-metastatic paradigm of protective stabilization. Highly expressed in pancreatic cancer, circPRKD3 directly binds the oncogenic RBP Musashi-2 (MSI2), protecting it from degradation by blocking β-TRCP-mediated ubiquitination. This upregulates focal adhesion-related genes (e.g., FLNA and ITGB1) and enhances cell migration and lung metastasis capability (48). This linear chain—“interaction → anti-ubiquitination → protein stabilization → focal adhesion remodeling”—clearly demonstrates how circRNAs can directly intervene in the post-translational fate of RBPs.

At the complex assembly level, circSATB1 exemplifies a scaffolding regulatory mode. In colorectal cancer, circSATB1 acts as a molecular scaffold, simultaneously binding the E3 ligase RNF25 and its substrate FKBP8, bringing them into spatial proximity to promote FKBP8 ubiquitination and degradation. FKBP8 is a natural inhibitor of mTOR signaling; its degradation leads to aberrant mTOR pathway activation, driving liver metastasis of tumor cells (49). Additionally, circRNAs can function through diverse modes, such as sequestering and inhibiting RBP function (e.g., circSMARCA5/SRSF1) and being regulated by RBPs during their biogenesis (RBM24/circ23679), collectively illustrating the broader regulatory diversity of circRNA–RBP interactions (50, 51). **Supplementary Table 2** provides a multi-column overview of reported circRNA–RBP interactions across tumor types, summarizing binding partners, downstream effectors, and functional roles.

### 3.3 Signaling pathway integration and EMT reprogramming: the common output of multiple mechanisms

Whether initiated through ceRNA networks or RBP interactions, the pro-metastatic effects of circRNAs require mediation via core signaling pathways to drive EMT transcriptional reprogramming. The TGF-β/Smad pathway, a core regulatory axis of EMT, represents a preferential target of circRNA action. circTGFBR2(3–6) provides a typical example: this circRNA, derived from back-splicing of TGFBR2 pre-mRNA, acts as a molecular scaffold in breast cancer cells. In an m^6^A-dependent manner, it recruits IGF2BP3 to TGFBR1 mRNA, enhancing its stability and sustaining TGF-β/Smad signaling activation, thereby driving EMT and cell migration (52, 53). Similarly, circRNAs regulate multiple pathways, including PI3K/AKT, Wnt/β-catenin, and Hippo, through mechanisms such as scaffold assembly, encoded peptides, or ceRNA activity, ultimately converging on activation of EMT transcription factors such as Snail and Zeb1 (54, 55). These factors, in turn, represent shared execution points for metastasis and immunosuppression, providing a mechanistic rationale for the hub circRNA concept.

### 3.4 Exosomal CircRNAs and metastatic niche formation: preconditioning the distant microenvironment

Exosomes, a subtype of extracellular vesicles (EVs), provide vehicles for the trans-organ delivery of circRNAs, enabling the pre-construction of a microenvironment conducive to metastatic colonization in distant organs before the arrival of tumor cells—the pre-metastatic niche (56). In esophageal squamous cell carcinoma, hypoxia significantly induces circ-ZNF609 enrichment in exosomes. Upon internalization by vascular endothelial cells, these exosomes upregulate VEGFA expression by sponging miR-150-5p to promote angiogenesis, while inhibiting the translation of tight junction proteins, such as ZO-1, by binding HuR, thereby enhancing vascular permeability. These two pathways collectively promote the formation of a pre-metastatic niche, facilitating vascular permeability and angiogenesis, thereby enabling distant dissemination of tumor cells (57). In liver metastasis, colorectal cancer cells release exosomes enriched with circ-0034880, which are taken up by hepatic macrophages, activating them into SPP1^high^CD206^+^ pro-metastatic macrophages and remodeling the liver stromal microenvironment into a niche permissive for metastatic colonization (58). In lymph node metastasis, circPDLIM5 is delivered to lymphatic endothelial cells via EVs, directly binding the transcription factor YY1 to enhance PROX1 expression, thereby driving lymphangiogenesis and lymph node metastasis (59).

### 3.5 Encoding functional peptides: expanding the translational dimension of metastasis regulation

Some circRNAs can transcend the non-coding boundary, initiating translation via IRES or m^6^A modification to generate functional peptides. In triple-negative breast cancer, the TRIM1-269aa protein translated from circTRIM1 enhances the binding of MARCKS to calmodulin, thereby promoting the calmodulin-dependent membrane translocation of MARCKS, activating the PI3K/AKT/mTOR pathway, and driving tumor metastasis and chemoresistance (34). This case reveals that circRNAs not only participate in metastasis through RNA-level regulation but can also directly intervene in signaling pathways at the protein level via their encoded products.

Moreover, some circRNAs have been confirmed to participate in metabolic reprogramming, influencing pathways such as glycolysis and lipid metabolism through ceRNA networks or protein interactions, thereby providing metabolic support for malignant tumor evolution (60, 61). The molecular mechanisms by which circRNAs promote tumor metastasis are summarized in **Figure 2**. The mechanisms by which circRNAs drive metastasis have been examined in this section, while the following section focuses on their roles in tumor immune evasion.

**Figure 2:**
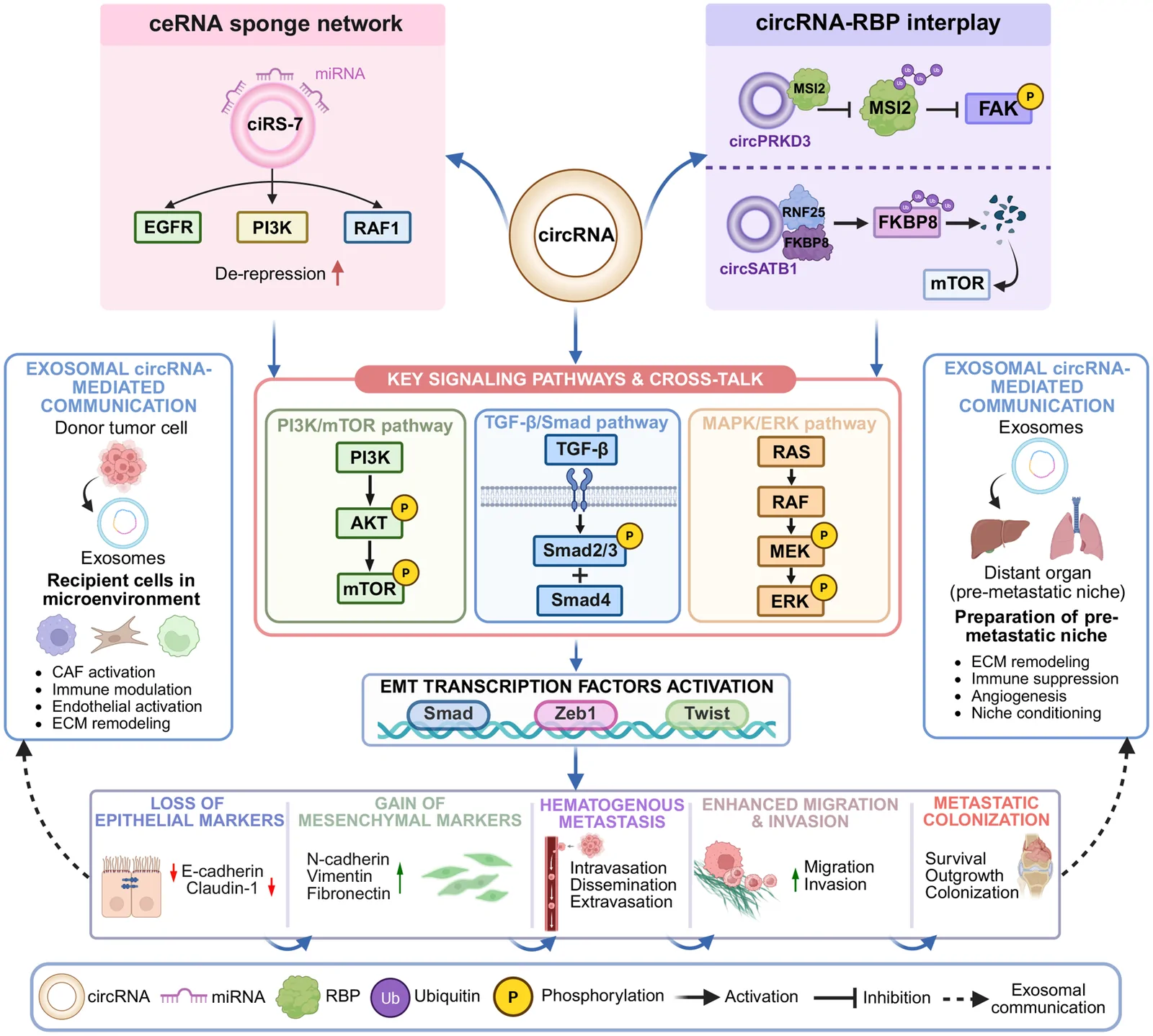
CircRNA-mediated mechanisms in tumor metastasis. CircRNAs promote metastasis through ceRNA networks, circRNA–RBP interactions, and exosome-mediated communication. These mechanisms activate PI3K/mTOR, TGF-β/Smad and MAPK/ERK pathways, leading to EMT, migration, invasion, hematogenous dissemination, and metastatic colonization. Created in BioRender. Zhao, Y (2026). https://BioRender.com/wdygzjt.

## 4 Pathways of CircRNA-mediated tumor immune evasion

Unlike the previous section, which is structured by molecular mechanisms, this section is organized according to functional consequences. The regulatory mechanisms involved, such as ceRNA networks and RBP interactions, largely overlap with those discussed in the metastasis section.

### 4.1 CircRNA regulation of immune checkpoints: multi-mechanism synergistic upregulation of PD-L1

The programmed death-1/programmed death-ligand 1 (PD-1/PD-L1) axis is a central checkpoint in tumor immune evasion, and circRNAs can upregulate PD-L1 expression via various molecular mechanisms (62). Circ‐CDYL exemplifies how a circRNA can upregulate PD‐L1 through direct protein interaction. In hepatocellular carcinoma, Circ-CDYL is significantly upregulated and directly binds the hornerin (HRNR) protein. By blocking SYVN1 E3 ubiquitin ligase-mediated HRNR ubiquitination and degradation, Circ-CDYL stabilizes HRNR protein levels. The accumulated HRNR further activates the mTORC1-p70S6K signaling axis, promoting PD-L1 transcription and translation. Peripheral blood mononuclear cell (PBMC) killing assays demonstrated that silencing Circ-CDYL significantly restored CD8+ T cell cytotoxicity and proliferation, while patient-derived organoids and humanized mouse models confirmed that activation of the Circ-CDYL/HRNR axis reduces the efficacy of anti-PD-L1 therapy. More critically, PD-L1+ exosomes released by Circ-CDYL-overexpressing tumor cells propagate immunosuppressive signals to other immune cells in the microenvironment, spatially amplifying the immune evasion effect (63). Beyond the aforementioned protein interaction pathways, circRNAs regulate PD-L1 through diverse mechanisms: in non-small cell lung cancer, circ_0000284 relieves PD-L1 mRNA inhibition by sponging miR-377-3p (64); in esophageal squamous cell carcinoma, CircNF1 promotes PD-L1 transcription via JAK-STAT3 pathway activation (65); in non-small cell lung cancer, CircENTPD7 upregulates PD-L1 by increasing IGF2BP2 expression (66).

Although the upstream pathways differ, PD‐L1 upregulation converges on a common outcome: suppression of T cell activity via PD‐1 engagement, thereby enabling the effector cell dysfunction discussed in the next section. Additionally, circRNAs have been reported to regulate other immune checkpoints, such as CTLA‐4, through analogous mechanisms, expanding the molecular repertoire of immune evasion (67, 68). **Supplementary Table 3** provides a multi-column summary of circRNAs involved in immune checkpoint regulation, detailing their molecular mechanisms and pathway involvement across different tumor types.

### 4.2 CircRNA-mediated impairment of anti-tumor immune effector cell function: from molecular inhibition to cellular dysfunction

Beyond upregulating immune checkpoints on tumor cells, circRNAs can directly impair the function of anti-tumor effector cells through diverse mechanisms. In adaptive immunity, CD8+ T cells are frequent targets of circRNA-mediated suppression. For instance, circRNA-002178 not only upregulates PD-L1 in tumor cells but also enters CD8+ T cells via exosomes, where it upregulates PD-1 expression, forming a “tumor PD-L1/T cell PD-1” bidirectional inhibitory axis that drives T cells into an exhausted state characterized by a marked decrease in the secretion of granzyme B, perforin, and interferon γ (IFN-γ) (69). Similarly, circCCAR1, after being internalized by T cells, further reinforces the maintenance of the exhausted phenotype by inhibiting ubiquitination-dependent degradation of PD-1 (70). At the innate immunity level, NK cells similarly undergo systemic suppression by circRNAs. Hepatocellular carcinoma cell-derived exosomal circUHRF1 upregulates TIM-3 expression on the NK cell surface by sponging miR-449c-5p, inducing NK cell functional exhaustion and impairing their ability to clear circulating tumor cells—this mechanism simultaneously amplifies both immune evasion and hematogenous metastasis (71). Furthermore, circRNAs can indirectly affect dendritic cell recruitment and antigen presentation efficiency by regulating chemokines, thereby impairing the priming of naïve T cells (72, 73). Notably, the exhausted T cells and functionally impaired NK cells described above not only lose tumor-killing capacity but may also coexist with regulatory immune-cell populations that secrete immunosuppressive cytokines such as IL-10 and TGF-β (74, 75). These factors help recruit and polarize immunosuppressive cells, thereby transforming a localized dysfunction into a niche-wide immunosuppressive environment.

### 4.3 CircRNA-mediated promotion of an immunosuppressive tumor microenvironment: from cellular dysfunction to niche remodeling

The functional impairment of effector cells does not represent the endpoint of immune evasion. Immunosuppressive cytokines secreted by regulatory immune cells and tumor cells further drive systemic remodeling of the TME. Among these processes, M2-type polarization of tumor-associated macrophages (TAMs) represents a core event in TME remodeling. Highly expressed in breast cancer, circCLASP1 directly binds the chaperonin CCT2, blocking E3 ubiquitin ligase-mediated degradation of GLI1, promoting GLI1 nuclear accumulation, and upregulating the transcription factor SNAIL. SNAIL activation, on one hand, drives the transcription and secretion of CCL2 and CCL5, recruiting macrophages and inducing their polarization toward the M2 phenotype; on the other hand, SNAIL itself is a core driver of EMT, thereby enabling circCLASP1 to enhance tumor metastatic potential while shaping an immunosuppressive microenvironment. Clinical data show that circCLASP1 is significantly upregulated in breast cancer tissues and serum, closely correlating with lymph node metastasis and poor prognosis (76). In addition to macrophage polarization, circRNAs may promote the differentiation and expansion of regulatory T cells by activating the TGF-β/Smad signaling pathway, enhance the intratumoral recruitment of myeloid-derived suppressor cells (MDSCs) through upregulation of chemokines such as CXCL12, and increase intratumoral regulatory T cells recruitment by upregulating chemokines such as CCL22 (77–79).

Collectively, these mechanisms transform immunosuppression from a transient cellular dysfunction into a stable, self-reinforcing niche characteristic. Once established, this niche not only shelters tumor cells from immune attack but also provides a favorable environment for metastatic outgrowth. Moreover, circRNAs can be packaged into exosomes, extending the immunosuppressive milieu beyond local boundaries.

### 4.4 Exosomal CircRNA-mediated transcellular immune regulation: propagation and amplification of immunosuppression

Tumor cells package circRNAs into exosomes and release them into the microenvironment and circulation. Upon internalization by T cells, NK cells, or macrophages, the delivered circRNAs reprogram the immune functions of recipient cells via mechanisms such as ceRNA sponging or signaling pathway activation, thereby spreading local immunosuppression into a systemic microenvironmental feature (80). Taken together with the roles of exosomal circRNAs in metastatic progression discussed in Section 3.4, these findings highlight exosome-mediated circRNA transfer as a common mode of intercellular communication involved in both metastasis and immune evasion (81).

From molecular checkpoint upregulation and effector cell dysfunction to immunosuppressive microenvironmental remodeling and exosomal propagation, circRNA-mediated immune evasion exhibits a clear, progressive spectrum. The sequential mechanisms by which circRNAs promote immune evasion are summarized in **Figure 3**. Notably, the recurring TGF-β, STAT3, and PD-L1 signaling axes are core pathways that drive metastasis as well—this molecular overlap strongly suggests the presence of hub circRNAs.

**Figure 3:**
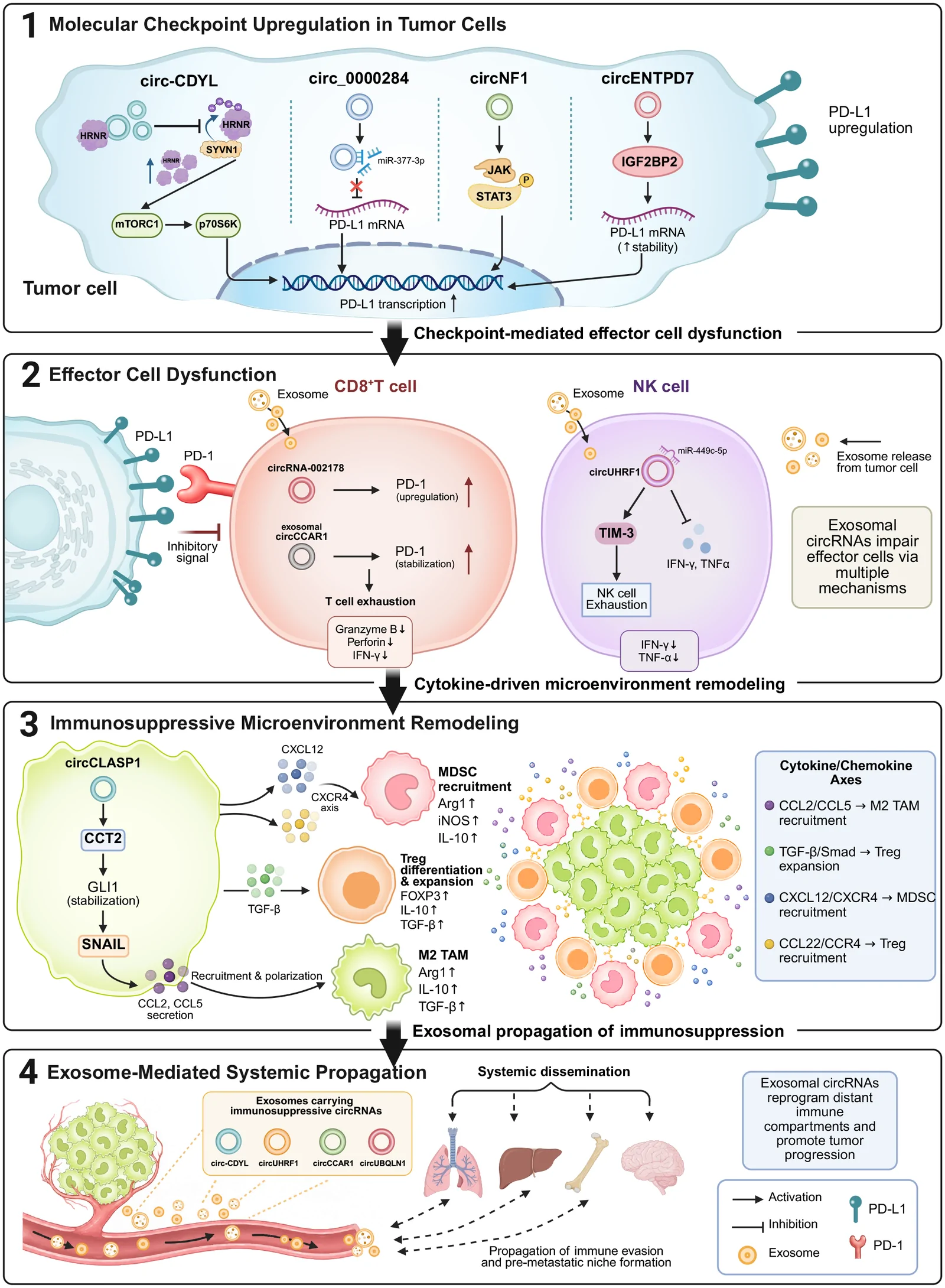
CircRNAs in tumor immune evasion. CircRNAs can help tumor cells escape immune attack by increasing checkpoint expression, weakening CD8+ T cell and NK cell activity, and reshaping the tumor microenvironment. Exosomal circRNAs further spread these effects, supporting M2 TAM polarization, Treg expansion, MDSC recruitment, and systemic immunosuppression. Created in BioRender. Zhao, Y (2026). https://BioRender.com/knwygh4.

## 5 Hub circRNAs: coordinated regulation of metastasis and immune evasion

The mechanisms of metastasis and immune evasion described in the preceding sections are not entirely separate. Their convergence at EMT-associated transcriptional programs, PD-L1 signaling, TGF-β/STAT3 activation, macrophage polarization, metabolic remodeling, and exosome-mediated communication highlights the existence of circRNAs that may functionally integrate metastatic and immune-evasion programs (16, 82). **Figure 4** summarizes the major regulatory mechanisms through which hub circRNAs coordinate metastasis and immune evasion within the same tumor context. The following sections first introduce the conceptual definition and evidence-mapping framework for hub circRNAs and then discuss their major synergistic regulatory mechanisms.

**Figure 4:**
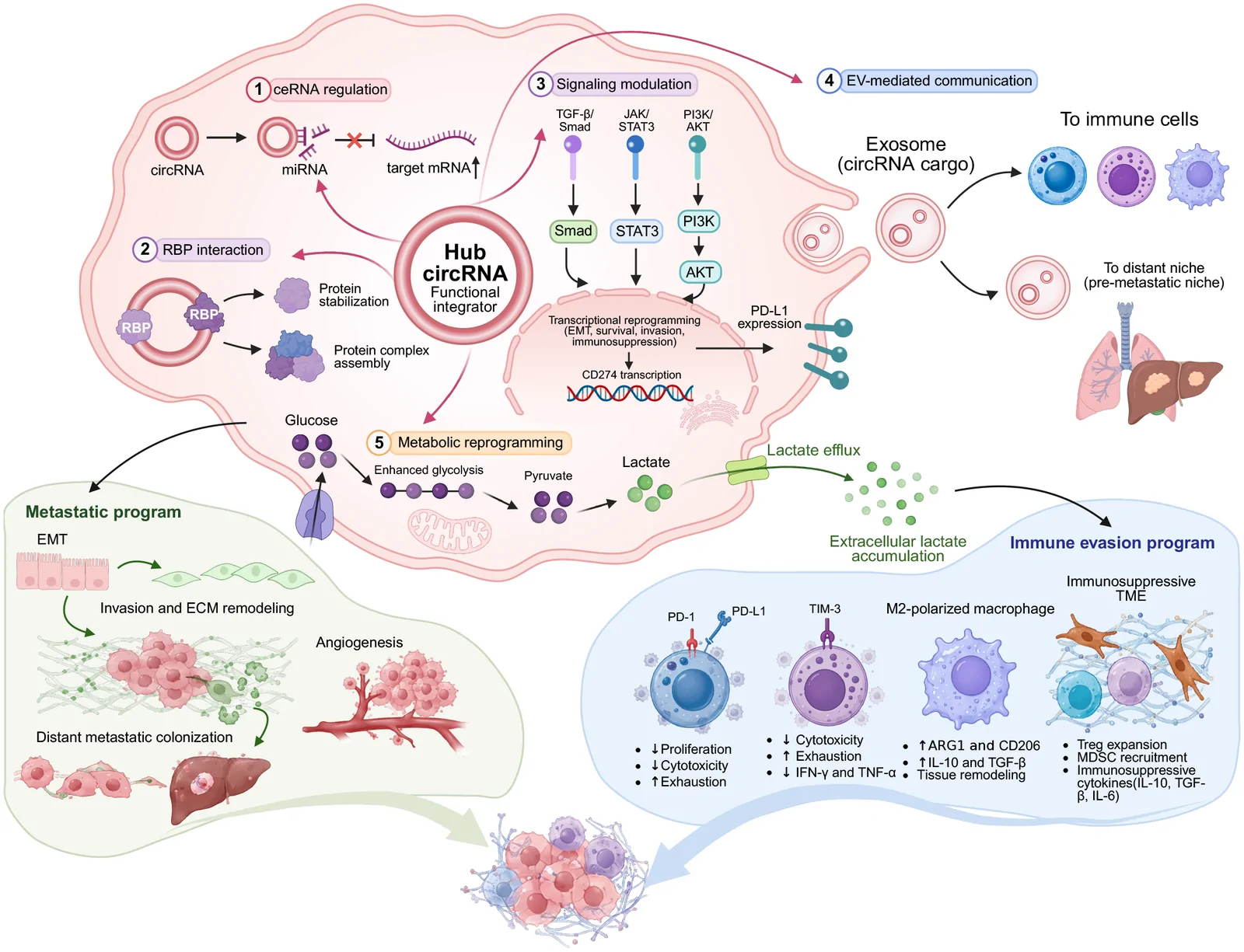
Multilayer functional integration mediated by hub circRNAs. Hub circRNAs coordinate metastasis and immune evasion through ceRNA regulation, RBP interactions, signaling modulation, extracellular vesicle-mediated communication, and metabolic reprogramming. These mechanisms promote metastatic progression while impairing antitumor immunity and reshaping the tumor microenvironment. Created in BioRender. Zhao, Y (2026). https://BioRender.com/9xkg9nk.

### 5.1 Conceptual definition and evidence-mapping framework of hub CircRNAs

Based on the biological convergence described above, we define “hub circRNAs” as circRNA molecules that functionally integrate metastatic and immune-evasion programs within the same tumor context. Unlike circRNAs that affect only a single malignant phenotype, hub circRNAs described in this framework are characterized by their capacity to coordinate multiple regulatory processes involved in both tumor dissemination and immune suppression. These regulatory effects may be mediated through diverse molecular layers, including ceRNA regulation, RNA-binding protein interactions, signaling pathway modulation, extracellular vesicle-mediated intercellular communication, and metabolic reprogramming. Thus, the defining feature of hub circRNAs in this framework is their ability to coordinate regulatory mechanisms that collectively influence both metastasis and immune evasion. Here, the term “hub” is used as a conceptual descriptor of functional integration rather than as a network-topological designation. This review does not reconstruct a unified regulatory network or calculate degree, betweenness, or other centrality metrics for individual circRNAs.

Accordingly, based on the available literature, we propose a three-tiered S/C/N evidence-mapping framework to organize current evidence according to its completeness and level of experimental support. **S-level (Stringent hubs)**: The same circRNA, within the same tumor context, possesses functional evidence for both metastasis and immune evasion, providing relatively complete dual-phenotype experimental evidence according to the criteria used in this review. **C-level (Convergent candidates)**: In a specific tumor context, functional evidence exists for either metastasis or immune evasion as a single phenotype, and mechanistic experiments confirm that the circRNA executes this function precisely through the regulation of recognized immune-metastasis intersection nodes, such as PD-L1/PD-1, TGF-β/STAT3, or M2-TAM polarization, thereby suggesting possible involvement in the other phenotype. However, functional validation of the circRNA in the other phenotype remains incomplete. **N-level (Network-informed candidates)**: In a specific tumor or pan-cancer analysis, bioinformatic approaches such as co-expression networks, ceRNA networks, or WGCNA indicate that the circRNA is associated with both metastasis-related and immune-related features. However, experimental causal evidence is lacking, and these circRNAs are included only as supplementary discussion.

Evidence assignment was conducted through literature-based evidence mapping. Relevant primary studies were identified from PubMed, Web of Science, and Google Scholar using terms related to circRNAs, cancer, metastasis, immune evasion, immunotherapy, and representative pathways or targets; reviews were used only to trace additional primary reports. Eligible studies were required to describe a specific circRNA in a defined tumor context. For S- and C-level assignment, the same tumor context referred to the same histological cancer type and, where relevant, a compatible molecular subtype. Evidence could be integrated across studies when the circRNA identity was unambiguously matched and the tumor context was concordant, although evidence generated within the same experimental system was considered stronger. Functional evidence required circRNA perturbation linked to phenotype-specific readouts: migration, invasion, metastatic burden, or distant colonization for metastasis; and altered immune-cell activity, checkpoint-dependent suppression, immunosuppressive-cell polarization or recruitment, or immunotherapy response for immune evasion. Mechanistic evidence required perturbation, binding, or rescue experiments linking the circRNA to the proposed molecular axis. Candidates were assigned sequentially: dual-phenotype functional evidence supported S-level designation; single-phenotype functional evidence plus mechanistic convergence supported C-level designation; and computational association with both phenotypes without causal validation supported N-level designation. CircRNAs with only expression or prognostic associations, insufficiently defined contexts, or no primary data were left unassigned. This process is summarized in **Supplementary Figure 1**.

**Supplementary Tables 4–6** compile circRNAs assigned to the S-, C-, and N-level evidence categories, providing detailed annotations on tumor types, functional evidence, mechanistic insights, and evidence sources. The following sections discuss representative hub circRNAs according to their major modes of coordinated regulation.

### 5.2 Intracellular synergy (I): single effector molecule dual-function type

The core strategy of intracellular synergy lies in a circRNA’s ability to synchronously activate metastasis and immune evasion programs within the same cell by regulating intracellular signaling networks, without leaving the tumor cell. The single-effector-molecule dual-function type focuses on a single key effector molecule inherently positioned at the endogenous intersection of metastasis and immune evasion signaling pathways. Based on how the dual function is achieved, two subtypes can be distinguished.

#### 5.2.1 Dependent on the effector molecule’s dual transcriptional regulatory activity

In this subtype, the effector molecule upregulated by the circRNA is a nuclear protein, such as a transcription factor or an epigenetic modifying enzyme. Upon entering the nucleus, it independently activates two sets of genetic programs: one drives EMT, conferring migratory and invasive capabilities, while the other directly binds to the promoter of an immune checkpoint gene to initiate its transcription.

In triple-negative breast cancer, highly expressed circC16orf62 upregulates the histone demethylase PHF8 through sponging miR-1299. PHF8 drives the EMT program to promote metastasis and concurrently upregulates *CD274* (encoding PD-L1), facilitating immune evasion. These two phenotypic programs operate in parallel, each separately initiated by the same effector molecule, PHF8, with both phenotypic effects supported by relatively complete experimental evidence in the same study (83). Similarly, in breast cancer, circLRBA blocks Twist1 ubiquitination and degradation by binding SPOP, stabilizing Twist1 to drive EMT while concurrently promoting PD-L1 transcription (84).

#### 5.2.2 Dependent on the effector molecule’s dual intrinsic function

In this subtype, the effector molecule regulated by the circRNA possesses dual intrinsic functions: executing immunosuppression and serving as a pro-metastatic signal source, with both functions operating in parallel. PD-L1 represents the most typical effector molecule.

circ_0089761, aberrantly expressed in colorectal cancer, serves as a representative example. circ_0089761 upregulates PD-L1 by sponging miR-27b-3p. This study reported that the upregulated PD-L1, on one hand, induces T cell exhaustion by binding to T cell PD-1 via its extracellular domain, facilitating immune evasion; on the other hand, it promotes metastasis by enhancing tumor cell migration and invasion (12). Mechanistically, another study indicated that the intracellular domain of PD-L1 can activate pro-migratory pathways such as PI3K/Akt and MAPK, suggesting that the two functions of PD-L1 may arise from distinct extracellular and intracellular activities of the same membrane protein, constituting a functional coupling of immune evasion and metastasis (85, 86). Beyond PD-L1, hsa_circ_0067842 in breast cancer enhances CMTM6 stability by affecting HuR nuclear translocation. CMTM6, in turn, promotes tumor cell migration and invasion while protecting PD-L1 from ubiquitination-dependent degradation (87).

### 5.3 Intracellular synergy (II): multi-effector pathway synergy type

Unlike the single-effector-molecule dual-function type, the multi-effector pathway synergy type does not rely on a single effector molecule. Instead, the circRNA engages multiple downstream effectors or pathways that collectively regulate metastasis and immune evasion. Based on the relationships among these downstream pathways, this type can be further classified into parallel-independent and cross-cascade subtypes.

#### 5.3.1 Dependent on multi-effector pathway parallel-independent regulation

These circRNAs simultaneously mobilize multiple downstream pathways to independently and in parallel drive metastasis and immune evasion, with no significant signaling intersection or upstream–downstream dependency between the two pathways.

circNCAPG, highly expressed in breast cancer, is a representative example of the parallel-independent type. A conference abstract reported that circNCAPG is localized in the cytoplasm and functions as a molecular sponge for miR-200b and miR-200c (miR-200s). miR-200s simultaneously target and inhibit the 3’UTRs of *ZEB-1* and *CSF-1*—the former is a core EMT transcription factor, and the latter is a macrophage chemokine. By sponging miR-200s, circNCAPG releases these two independent pathways in parallel: it upregulates *ZEB-1*, driving the EMT program and conferring migratory and invasive capabilities to tumor cells, while simultaneously promoting *CSF-1* secretion, recruiting tumor-associated macrophages to shape an immunosuppressive microenvironment. Upon circNCAPG knockdown, expression of both *CSF-1* and *ZEB-1* decreased simultaneously, and the metastatic phenotype was suppressed (88). This paradigm illustrates a representative regulatory logic: “one upstream switch → two parallel-independent pathways.”

#### 5.3.2 Dependent on multi-effector pathway cross-cascade regulation

Unlike the parallel-independent type, in which downstream pathways act relatively independently, cross-cascade regulation occurs when one circRNA-activated downstream program lies upstream of and promotes another, thereby establishing a sequential mechanistic connection between metastasis and immune evasion.

circNSD2, which is induced by TGF-β in triple-negative breast cancer, provides a representative example of cross-cascade regulation. Mechanistically, circNSD2 functions as a molecular scaffold that enhances the interaction between USP10 and SRSF6, thereby reducing K48-linked polyubiquitination and proteasomal degradation of SRSF6. Stabilized SRSF6 reprograms TPM1 alternative splicing, promoting EMT, tumor cell migration, invasion, and metastasis. Importantly, this TPM1 alternative-splicing program also increases PD-L1 expression through Twist-dependent transcription, suppresses CD8+ T-cell-mediated antitumor activity, and facilitates immune evasion. Thus, circNSD2 links a metastasis-associated alternative-splicing program to PD-L1-mediated immunosuppression through a sequential cross-cascade within the same tumor context (89).

### 5.4 Extracellular synergy: remote regulation by exosomal CircRNAs

The hallmark of the extracellular synergy strategy is that circRNAs are packaged into exosomes and secreted from the cell, indirectly promoting metastasis and immune evasion by remotely regulating other cells. Based on spatial distance and target, this can be classified into local microenvironment remodeling and distant pre-metastatic niche formation.

#### 5.4.1 Local microenvironment remodeling

In this subtype, tumor-derived exosomes act locally within the primary tumor stroma. The circRNAs they carry are taken up by intratumoral immune cells, reprogramming them into an immunosuppressive phenotype and thereby remodeling the local immune microenvironment to favor tumor progression.

In non-small cell lung cancer, hypoxia-induced circPLEKHM1 enters tumor-associated macrophages via exosomes, driving their polarization toward the M2 phenotype. These reprogrammed M2 macrophages not only highly express and secrete immunosuppressive factors such as IL-10 and TGF-β but also thereby establish an immunosuppressive phenotype. Meanwhile, paracrine factors such as TGF-β feed back onto tumor cells, enhancing EMT (as indicated by upregulated protein levels of N-cadherin and vimentin) and tumor cell migration and invasion. This study provided experimental evidence within the same system supporting both M2 macrophage polarization and enhanced tumor cell metastatic capacity, thereby forming a “tumor → immune cell → tumor” local vicious cycle (90). Similarly, exosomal circSAFB2 induces M2 macrophage polarization via the miR-620/JAK1/STAT3 axis, concurrently impairing antitumor immunity and enhancing tumor cell invasiveness (91).

#### 5.4.2 Distant pre-metastatic niche formation

A more proactive strategy involves tumors releasing circRNA-carrying exosomes into the circulation as early systemic signals before overt metastasis occurs. These exosomes travel through the bloodstream to target organs and are internalized by resident macrophages, pre-shaping an immunosuppressive microenvironment conducive to tumor cell colonization.

circ-0034880 in colorectal cancer liver metastasis serves as a representative example: exosomes released by primary colorectal cancer cells reach the liver via the blood circulation. After internalization by liver macrophages, they upregulate *SPP1* expression by acting as molecular sponges for miR-200a-3p and miR-141-3p, activating them into SPP1^high^CD206^+^ pro-tumor macrophages. This activation process remodels the liver stromal microenvironment, including promoting α-SMA^+^ fibroblast infiltration, creating a “soil” conducive to tumor cell colonization before the formation of macroscopic metastases. Subsequently, arriving tumor cells efficiently colonize this microenvironment. This study provided animal-model evidence supporting the pro-metastatic effect of this axis and its potential feasibility as an interventional target (58). **Supplementary Table 7** summarizes circRNAs packaged into exosomes, detailing their source cells, target cells, functional effects, and mechanistic annotations across tumor types.

### 5.5 Metabolism-mediated synergy: the dual-fueling effect of metabolic reprogramming

The preceding sections were organized sequentially, progressing from intracellular to extracellular synergy modes. Recent studies indicate that circRNAs can also exert synergistic effects via metabolic reprogramming. The lactate and other metabolites they produce not only promote tumor cell invasion and migration *in situ* but also diffuse into the microenvironment to modulate immune cell functions, highlighting a noteworthy emerging frontier (92, 93).

Through metabolic reprogramming, these circRNAs allow key metabolites to support both metastatic progression and immune evasion. In lung adenocarcinoma cells, circP4HB binds pyruvate kinase M2, enhancing its tetramer formation and pyruvate kinase activity, thereby driving aerobic glycolysis and promoting lactate accumulation. This process not only supplies ATP and biosynthetic precursors for cell migration and invasion (significantly correlating with lymph node metastasis and high TNM stage) but also induces tumor-associated macrophages to polarize toward the M2 phenotype by targeting pyruvate kinase M2, supporting a potential dual role in promoting metastatic behavior and immune evasion (94). In non-small cell lung cancer, circRUNX1 upregulates HK2 by sponging miR-145, enhancing the Warburg effect and lactate production. This promotes tumor proliferation and metastasis and drives immune evasion by promoting infiltration of regulatory T cells via lactate accumulation (95). In hepatocellular carcinoma, the encoded peptide circPETH-147aa also promotes glycolysis and metastasis while inhibiting CD8+ T cell function (96). Whether these metabolites can achieve dual-phenotype causal verification within the same system remains a key bottleneck awaiting breakthroughs in this area (60).

The coordinated regulation of metastasis and immune evasion by hub circRNAs is summarized in **Figure 5**.

**Figure 5:**
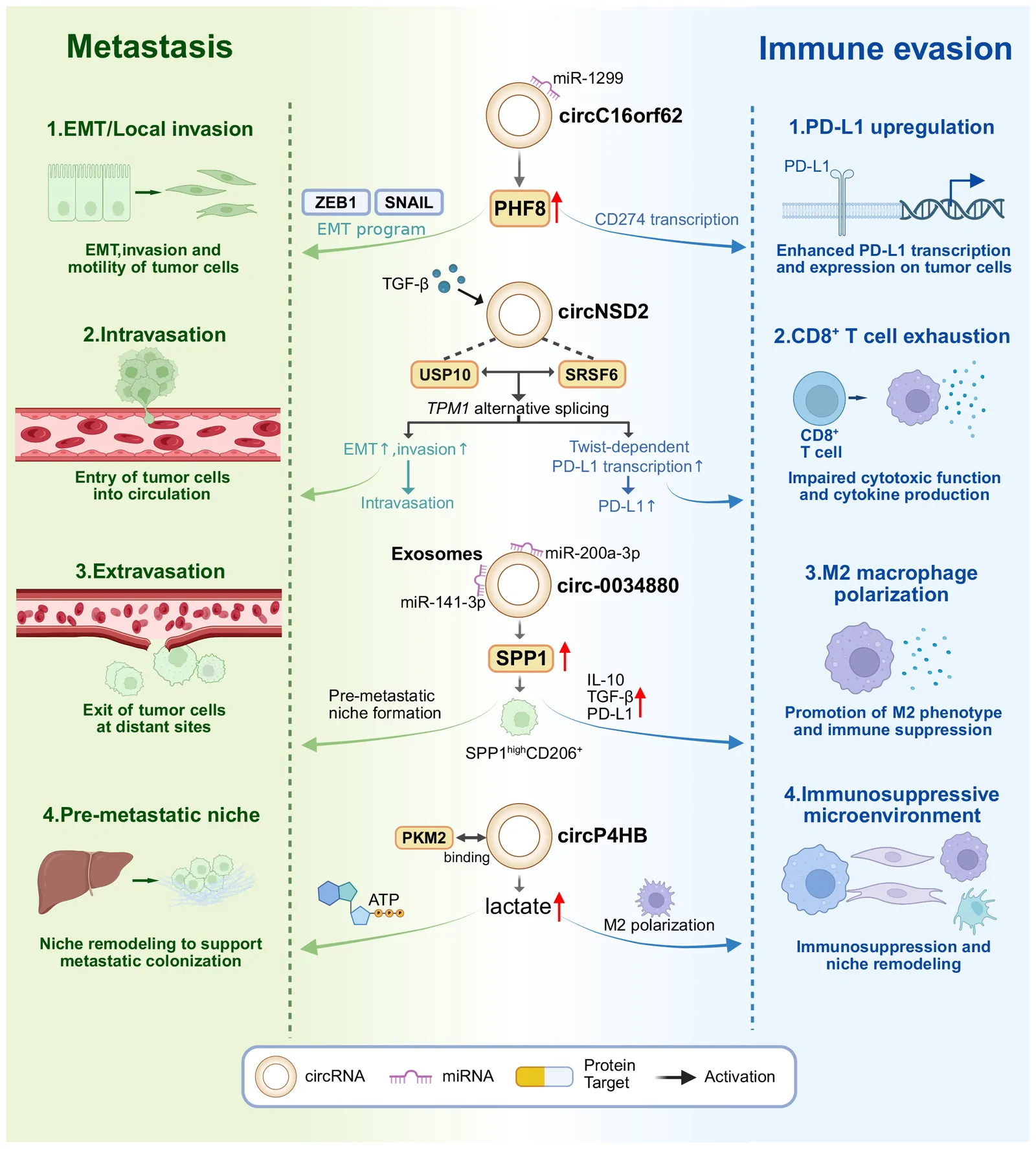
Hub circRNAs linking metastasis and immune evasion. Representative hub circRNAs are shown as examples of how one circRNA-related axis can connect metastatic behavior with immune evasion in the same tumor setting. circC16orf62, circNSD2, circ-0034880, and circP4HB highlight transcriptional regulation, protein stabilization and alternative splicing, exosome-mediated niche remodeling, and metabolism-linked immunosuppression. Created in BioRender. Zhao, Y (2026). https://BioRender.com/wdygzjt.

## 6 Discussion

The pro-metastatic functions and immunomodulatory roles of circRNAs have often been discussed separately in previous studies, and researchers have not yet commonly integrated them into a unified framework. However, the recurring emergence of signaling nodes such as TGF-β, STAT3, and PD-L1 in both malignant phenotypes indicates that these two processes exhibit clear molecular intersections rather than being entirely independent. This mechanistic overlap suggests that simultaneously targeting both malignant programs may produce a therapeutic effect greater than targeting either program alone. In light of this, this review proposes the concept of “hub circRNAs” to refer to circRNAs that can simultaneously coordinate metastasis and immune evasion. It also adopts a synergistic regulatory perspective to compensate for the previous research orientation, which has been skewed toward single phenotypes.

Building on this perspective, this review outlines a provisional S/C/N evidence-mapping framework and organizes circRNAs according to the available evidence and their proposed functional synergy modes. The core issue addressed by this framework is that when a single circRNA simultaneously participates in both metastasis and immune evasion, functional interpretations based solely on single-phenotype readouts are likely to underestimate—or even miss—its pathological significance. Supporting hub circRNA designation, therefore, requires completing a dual-phenotype causal verification loop—covering both metastasis and immune evasion—within the same tumor context. This implies that experimental designs must shift from single- to dual-endpoint evaluation, imposing more stringent methodological demands on animal models (e.g., systems that combine immune competence with trackable metastatic capacity), on the dimensions of functional readouts, and on cross-study comparability.

However, this framework should currently be viewed as a literature‐based organizing tool rather than an externally validated standard, a cautious stance warranted by several interconnected limitations. First, the framework’s categories are inevitably shaped by the uneven and incomplete literature, with few studies that concurrently address metastasis and immune evasion in the same tumor context, thereby limiting the number of S‐level examples and signaling the immaturity of the evidence base. Second, cross‐study comparisons are confounded by substantial heterogeneity in experimental models, functional assays, validation depth, and cancer types, and the framework does not yet offer a standardized procedure for adjudicating discrepant or incomplete findings. Third, most representative candidate hub circRNAs discussed here are supported by single mechanistic studies, necessitating independent replication, and their clinical relevance to immunotherapy remains largely unvalidated—whether they can predict responses to anti‐PD‐1, anti‐PD‐L1, or anti‐CTLA‐4 therapy, contribute to primary or acquired resistance, or improve checkpoint inhibitor sensitivity when the circRNA is inhibited—all of which await testing in clinical cohorts and treatment‐response models. Consequently, future efforts must prioritize prospective testing, independent datasets, standardized evidence-evaluation procedures, and broader scrutiny by the circRNA research community, as well as validation across diverse preclinical and clinical systems to establish the framework’s practical utility.

On this basis, several critical research gaps remain in this field and urgently need to be addressed. First, nomenclature and database annotation inconsistency remain major barriers. The limited overlap in circRNA annotations and the lack of unified naming conventions across existing databases hinder systematic screening and cross-study comparison of candidate hub circRNAs. Second, experimental methodology poses substantial challenges. On one hand, circRNA identification can be confounded by reverse-transcription template switching, RNA-seq mapping artifacts, and incomplete discrimination from linear host transcripts (97, 98); on the other hand, functional studies often rely on a single siRNA/shRNA without rescue experiments, weakening causal attribution of phenotypes to specific circRNAs (97, 99). Moreover, the vast majority of *in vivo* studies use immunodeficient mouse models, which cannot simultaneously evaluate metastatic burden and immune microenvironment remodeling within the same animal, forcing researchers to choose between immune‐competent models and metastasis‐trackable models—a direct conflict with the intrinsic requirement for dual‐phenotype closed‐loop validation of hub circRNAs. Third, the total number of studies concurrently investigating both metastasis and immune evasion remains limited, and the currently available S‐level examples cover only a few cancer types, leaving pan‐cancer generalizability unestablished. Fourth, metabolic reprogramming, as a rapidly emerging dimension, is still predominantly studied through single-phenotype observations among candidate hub circRNAs. Whether the two lines of evidence—metabolite‐driven metastasis and metabolite‐driven immunosuppression—can converge into a closed‐loop causal chain within the same experimental system remains a core question awaiting verification.

Addressing these gaps will expand the translational potential of this field. The S/C/N framework serves as a practical evidence-mapping tool. Assigning molecules to stringent S-level hubs, convergent C-level candidates, and network-informed N-level candidates helps avoid conflating causal functional validation with bioinformatic association. At the same time, this framework can assist in prioritizing molecules for future validation, thereby facilitating more focused progress in translational and mechanistic research. In clinical translation, the core value of hub circRNAs lies not in listing more candidate molecules, but in converting their dual capacity to drive both metastasis and immune evasion into tools that are detectable, stratifiable, and actionable. Regarding biomarkers and liquid biopsy, the most readily implementable approach is the construction of “metastasis–immune evasion combined risk scores.” Several S-level hubs identified in this review—including circLRBA, hsa_circ_0067842, circC16orf62, and circ_0089761—have been linked to EMT activation, PD-L1 upregulation, or immune evasion in relevant cohorts, suggesting their potential as composite biomarkers that complement PD-L1, MSI or TMB, and ctDNA residual disease markers (100, 101). The high stability and repeat-sampling advantages of circRNAs in plasma, serum, and exosomes are driving such strategies from retrospective discovery toward prospective validation (102). In therapeutic intervention, the most promising strategy centers on precise silencing of the back-splice junction using ASOs, siRNAs, or CRISPR-Cas13 (103). Its theoretical advantage lies in the ability to simultaneously interfere with both metastasis-related programs—including EMT, invasion, and distant colonization—and immune-evasion modules—including PD-L1 upregulation, macrophage polarization, and immunosuppressive microenvironment remodeling. This dual-action capacity distinguishes hub circRNA targeting from conventional single-axis agents, such as EMT inhibitors or immune checkpoint blockers, which address only one side of this interconnected malignant circuitry. By disrupting both processes concurrently, hub-directed strategies may interrupt their reciprocal crosstalk and potentially yield more durable therapeutic responses than approaches targeting either pathway alone. Combination regimens align more closely with the biological logic of hub circRNAs than monotherapy, and future efforts may prioritize exploring the synergy between anti‐PD‐1/PD‐L1 agents and circRNA inhibitors or CXCR4/TGF‐β/STAT3 pathway inhibitors (104, 105). Beyond direct RNA interference, circRNA-encoded cryptic peptides have also demonstrated clear immunogenicity. The cryptic peptide encoded by circFAM53B, for instance, can effectively prime CD4+ and CD8+ T cells and induce antitumor immunity. An mRNA vaccine based on this finding has since entered an early-phase clinical trial (NCT07245901), highlighting its potential as a tumor-specific vaccine antigen, although its therapeutic efficacy and the patient populations most likely to benefit remain to be defined in larger studies (106). In clinical advancement, subsequent research must shift from demonstrating relevance to demonstrating utility. Pre-analytical processing, exosome characterization, and quantitative reporting should first be standardized according to the MISEV2023 guidelines together with the MIQE/dMIQE standards. This should be followed by multicenter studies that systematically collect paired tissue, plasma, and exosome samples from the same patients, with prespecified thresholds independently validated against drug response and recurrence endpoints to establish clinical utility and lay the foundation for future companion-diagnostics co-development (107–110). Ultimately, the translational promise of hub circRNAs rests on the recognition that metastasis and immune evasion are not separate challenges to be tackled in isolation, but interconnected facets of tumor progression that hub circRNAs naturally coordinate—and that may be most effectively disrupted as a unified axis.

## Supplementary material

Supplementary Figure 1 A provisional workflow for S/C/N evidence assignment of candidate hub circRNAs. Candidate circRNAs are sequentially evaluated according to dual-phenotype functional evidence, single-phenotype functional evidence with mechanistic convergence, and network-informed association. Candidates that do not meet any of these criteria remain unassigned. Created in BioRender. Zhao, Y (2026). https://BioRender.com/gngn0gd.

## References

[1] Biswas J Boussi L Stein E Abdel-Wahab O . Aberrant pre-mRNA processing in cancer. J Exp Med. (2024) 221. doi: 10.1084/jem.20230891 39316554 PMC11448470

[2] Anczukow O Allain F Angarola B Black D Brooks A Cheng C . Steering research on mRNA splicing in cancer towards clinical translation. Nat Rev Cancer. (2024) 24:887–905. doi: 10.1038/s41568-024-00750-2 39384951 PMC11698124

[3] Gimeno-Valiente F López-Rodas G Castillo J Franco L . The many roads from alternative splicing to cancer: Molecular mechanisms involving driver genes. Cancers (Basel). (2024) 16. doi: 10.3390/cancers16112123 38893242 PMC11171328

[4] Pan H Wu X Hu B Xiong H Luo Y Zhou J . Nonsense-mediated mRNA decay: a key regulatory system engaged in cancer. Cell Commun Signal. (2025) 23:489. doi: 10.1186/s12964-025-02503-6 41219960 PMC12607236

[5] Ebbesen K Hansen T Kjems J . Insights into circular RNA biology. RNA Biol. (2017) 14:1035–45. doi: 10.1080/15476286.2016.1271524 27982727 PMC5680708

[6] Nisar S Bhat A Singh M Karedath T Rizwan A Hashem S . Insights into the role of CircRNAs: Biogenesis, characterization, functional, and clinical impact in human Malignancies. Front Cell Dev Biol. (2021) 9:617281. doi: 10.3389/fcell.2021.617281 33614648 PMC7894079

[7] Yu C Kuo H . The emerging roles and functions of circular RNAs and their generation. J BioMed Sci. (2019) 26:29. doi: 10.1186/s12929-019-0523-z 31027496 PMC6485060

[8] Feng X Zhu S Pu K Huang H Chen Y Wang W . New insight into circRNAs: characterization, strategies, and biomedical applications. Exp Hematol Oncol. (2023) 12:91. doi: 10.1186/s40164-023-00451-w 37828589 PMC10568798

[9] Hashemi M Khosroshahi E Daneii P Hassanpoor A Eslami M Koohpar Z . Emerging roles of CircRNA-miRNA networks in cancer development and therapeutic response. Non-coding RNA Res. (2025) 10:98–115. doi: 10.1016/j.ncrna.2024.09.006 39351450 PMC11440256

[10] Zhang N Wang X Li Y Lu Y Sheng C Sun Y . Mechanisms and therapeutic implications of gene expression regulation by circRNA-protein interactions in cancer. Commun Biol. (2025) 8:77. doi: 10.1038/s42003-024-07383-z 39825074 PMC11748638

[11] Ding Z Luo Z Zhang L Zhang S Zhang R Qiu Z . State-of-the-art strategies for circular RNA in cancers: Opportunity and challenge. MedComm (2020). (2026) 7:e70608. doi: 10.1002/mco2.70608 41664705 PMC12883039

[12] Gao Q Cheng X Gao X . Circ_0089761 accelerates colorectal cancer metastasis and immune escape via miR-27b-3p/PD-L1 axis. Physiol Rep. (2024) 12:e70137. doi: 10.14814/phy2.70137 39632246 PMC11617067

[13] Lv Z Xie G Xu W . Toward precision oncology: deciphering the circRNA-EMT axis in cancer and its therapeutic implications. RNA Biol. (2026) 23:1–24. doi: 10.1080/15476286.2026.2639611 41949207 PMC13078205

[14] Sasi N Preetha D Iyyappan S Selvamurugan N . Circular RNAs: Emerging regulators of signaling pathways in epithelial-mesenchymal transition and angiogenesis during breast cancer progression. Semin Oncol. (2025) 52:152340. doi: 10.1016/j.seminoncol.2025.152340 40220369

[15] Sanati M Ghafouri-Fard S . Circular RNAs: key players in tumor immune evasion. Mol Cell Biochem. (2025) 480:3267–95. doi: 10.1007/s11010-024-05186-8 39754640

[16] Yang K Zhang Y Xiong J Ai B Han D Chong X . Novel insights into the role of circRNAs in cancer immunotherapy resistance and clinical implications. Int J Mol Sci. (2026) 27. doi: 10.3390/ijms27083678 42074315 PMC13116664

[17] Guo Y Gong Y Wu M Ji M Xie F Chen H . CircRNAs in the tumor microenvironment: new frontiers in cancer progression and therapy. Crit Rev Oncol Hematol. (2025) 212:104754. doi: 10.1016/j.critrevonc.2025.104754 40320223

[18] Wang Y Dzakah E Wang X . Molecular basis of backsplicing regulation and its application to manipulate circRNA levels. RNA Biol. (2025) 22:1–10. doi: 10.1080/15476286.2025.2525914 40575920 PMC12218527

[19] Kristensen L Jakobsen T Hager H Kjems J . The emerging roles of circRNAs in cancer and oncology. Nat Rev Clin Oncol. (2022) 19:188–206. doi: 10.1038/s41571-021-00585-y 34912049

[20] Zhang X Wang H Zhang Y Lu X Chen L Yang L . Complementary sequence-mediated exon circularization. Cell. (2014) 159:134–47. doi: 10.1016/j.cell.2014.09.001 25242744

[21] Yang Y Zhong Y Chen L . EIciRNAs in focus: current understanding and future perspectives. RNA Biol. (2025) 22:1–12. doi: 10.1080/15476286.2024.2443876 39711231 PMC12710894

[22] Zhong Y Yang Y Wang X Ren B Wang X Shan G . Systematic identification and characterization of exon-intron circRNAs. Genome Res. (2024) 34:376–93. doi: 10.1101/gr.278590.123 38609186 PMC11067877

[23] Ares M Jr. Igel H Katzman S Donohue J . Intron lariat spliceosomes convert lariats to true circles: implications for intron transposition. Genes Dev. (2024) 38:322–35. doi: 10.1101/gad.351764.124 38724209 PMC11146597

[24] Wang X Shan G . Features and biomedical relevance of circular RNA biogenesis. Trends Genet. (2026) 42:282–94. doi: 10.1016/j.tig.2025.11.001 41352990

[25] Hwang H Kim Y . Molecular mechanisms of circular RNA translation. Exp Mol Med. (2024) 56:1272–80. doi: 10.1038/s12276-024-01220-3 38871818 PMC11263353

[26] Xiao J Joseph S Xia M Teng F Chen X Huang R . Circular RNAs acting as miRNAs' sponges and their roles in stem cells. J Clin Med. (2022) 11. doi: 10.3390/jcm11102909 35629034 PMC9145679

[27] Conn V Chinnaiyan A Conn S . Circular RNA in cancer. Nat Rev Cancer. (2024) 24:597–613. doi: 10.1038/s41568-024-00721-7 39075222

[28] Pisignano G Michael D Visal T Pirlog R Ladomery M Calin G . Going circular: history, present, and future of circRNAs in cancer. Oncogene. (2023) 42:2783–800. doi: 10.1038/s41388-023-02780-w 37587333 PMC10504067

[29] Huang C Liang D Tatomer D Wilusz J . A length-dependent evolutionarily conserved pathway controls nuclear export of circular RNAs. Genes Dev. (2018) 32:639–44. doi: 10.1101/gad.314856.118 29773557 PMC6004072

[30] Li J Wang X . Functional roles of conserved lncRNAs and circRNAs in eukaryotes. Non-coding RNA Res. (2024) 9:1271–9. doi: 10.1016/j.ncrna.2024.06.014 39036601 PMC11260338

[31] Chen L Shan G . CircRNA in cancer: Fundamental mechanism and clinical potential. Cancer Lett. (2021) 505:49–57. doi: 10.1016/j.canlet.2021.02.004 33609610

[32] Chakravarthi V Christenson L . Wet lab techniques for the functional analysis of circular RNA. Cells. (2025) 14. doi: 10.3390/cells14231920 41369410 PMC12691094

[33] Song X Wei Z Zhang C Han D Liu J Song Y . CircAKT3 promotes prostate cancer proliferation and metastasis by enhancing the binding of RPS27A and RPL11. Mol Cancer. (2025) 24:53. doi: 10.1186/s12943-025-02261-6 39994725 PMC11852832

[34] Li Y Wang Z Yang J Sun Y He Y Wang Y . CircTRIM1 encodes TRIM1-269aa to promote chemoresistance and metastasis of TNBC via enhancing CaM-dependent MARCKS translocation and PI3K/AKT/mTOR activation. Mol Cancer. (2024) 23:102. doi: 10.1186/s12943-024-02019-6 38755678 PMC11097450

[35] Liu Z Chen J Ren Y Liu S Ba Y Zuo A . Multi-stage mechanisms of tumor metastasis and therapeutic strategies. Signal Transduct Target Ther. (2024) 9:270. doi: 10.1038/s41392-024-01955-5 39389953 PMC11467208

[36] Fang L Zhu Z Han M Li S Kong X Yang L . Unlocking the potential of extracellular vesicle circRNAs in breast cancer: From molecular mechanisms to therapeutic horizons. BioMed Pharmacother. (2024) 180:117480. doi: 10.1016/j.biopha.2024.117480 39357330

[37] Chen J Yang J Fei X Wang X Wang K . CircRNA ciRS-7: a novel oncogene in multiple cancers. Int J Biol Sci. (2021) 17:379–89. doi: 10.7150/ijbs.54292 33390857 PMC7757028

[38] Zhang Y Xiong C Jiang Z Wang X Ji J Pan Y . Circular RNA CDR1as/ciRS-7- a novel biomarker in solid tumors. Front Oncol. (2024) 14:1468363. doi: 10.3389/fonc.2024.1468363 39678511 PMC11638042

[39] Ghafouri-Fard S Khoshbakht T Hussen B Sarfaraz S Taheri M Ayatollahi S . Circ_CDR1as: a circular RNA with roles in the carcinogenesis. Pathol Res Pract. (2022) 236:153968. doi: 10.1016/j.prp.2022.153968 35667198

[40] Xu B Yang T Wang Z Zhang Y Liu S Shen M . CircRNA CDR1as/miR-7 signals promote tumor growth of osteosarcoma with a potential therapeutic and diagnostic value. Cancer Manag Res. (2018) 10:4871–80. doi: 10.2147/cmar.S178213 30425578 PMC6203089

[41] Zhang Y Stuelten C . Alternative splicing in EMT and TGF-β signaling during cancer progression. Semin Cancer Biol. (2024) 101:1–11. doi: 10.1016/j.semcancer.2024.04.001 38614376 PMC11180579

[42] Meng L Liu S Ding P Chang S Sang M . Circular RNA ciRS-7 inhibits autophagy of ESCC cells by functioning as miR-1299 sponge to target EGFR signaling. J Cell Biochem. (2020) 121:1039–49. doi: 10.1002/jcb.29339 31490018

[43] Dou Z Gao L Ren W Zhang H Wang X Li S . CiRS-7 functions as a ceRNA of RAF-1/PIK3CD to promote metastatic progression of oral squamous cell carcinoma via MAPK/AKT signaling pathways. Exp Cell Res. (2020) 396:112290. doi: 10.1016/j.yexcr.2020.112290 32956704

[44] Zhong G Zhao Q Chen Z Yao T . TGF-β signaling promotes cervical cancer metastasis via CDR1as. Mol Cancer. (2023) 22:66. doi: 10.1186/s12943-023-01743-9 37004067 PMC10064584

[45] Feng Y Yang F Zheng J Shi L Xie T Lin Y . Circular RNA HIPK3 mediates epithelial-mesenchymal transition of retinal pigment epithelial cells by sponging multiple microRNAs. Sci Rep. (2024) 14:28872. doi: 10.1038/s41598-024-71119-6 39572643 PMC11582593

[46] Ghafouri-Fard S Khoshbakht T Taheri M Jamali E . A concise review on the role of CircPVT1 in tumorigenesis, drug sensitivity, and cancer prognosis. Front Oncol. (2021) 11:762960. doi: 10.3389/fonc.2021.762960 34804965 PMC8599443

[47] Yang L Li C Jiang X Yuan Y Li C Zhou Q . Functional roles and mechanisms of circRNA-protein interactions in cancer progression and tumor immune regulation. Front Immunol. (2026) 17:1771949. doi: 10.3389/fimmu.2026.1771949 41756268 PMC12932422

[48] Li Z Gao L Yang Y Ming Y Liu W Zhang T . A circular RNA promotes tumor metastasis through stabilizing MSI2 protein in pancreatic ductal adenocarcinoma. Res (Wash D C). (2025) 8:918. doi: 10.34133/research.0918 41049614 PMC12491784

[49] Zhang C Tian C Zhu R Chen C Jin C Wang X . CircSATB1 promotes colorectal cancer liver metastasis through facilitating FKBP8 degradation via RNF25-mediated ubiquitination. Adv Sci (Weinh). (2025) 12:e2406962. doi: 10.1002/advs.202406962 39921520 PMC11967755

[50] Barbagallo D Caponnetto A Barbagallo C Battaglia R Mirabella F Brex D . The GAUGAA motif is responsible for the binding between circSMARCA5 and SRSF1 and related downstream effects on glioblastoma multiforme cell migration and angiogenic potential. Int J Mol Sci. (2021) 22. doi: 10.3390/ijms22041678 33562358 PMC7915938

[51] Liu J Yao D Zhou F Li K Sun W Wang S . RBM24-mediated biogenesis of circ23679 protects cardiomyocytes against apoptosis via sponging miR-15b-5p. Int J Biol Macromol. (2025) 310:143242. doi: 10.1016/j.ijbiomac.2025.143242 40250678

[52] Wang Q Tjokrodirijo RTN Mei H van Veelen PA Ten Dijke P Fan C . circTGFBR2(3-6) acts as an assembly platform for RNA-binding protein IGF2BP3 and TGFBR1 mRNA to enhance breast cancer cell plasticity. Cell Death Differ. (2026) 33:779–97. doi: 10.1038/s41418-025-01597-2 41145815 PMC13077047

[53] Li K Guo J Ming Y Chen S Zhang T Ma H . A circular RNA activated by TGFβ promotes tumor metastasis through enhancing IGF2BP3-mediated PDPN mRNA stability. Nat Commun. (2023) 14:6876. doi: 10.1038/s41467-023-42571-1 37898647 PMC10613289

[54] Wang S Cheng L Wu H Li G . Mechanisms and prospects of circular RNAs and their interacting signaling pathways in colorectal cancer. Front Oncol. (2022) 12:949656. doi: 10.3389/fonc.2022.949656 35992800 PMC9382640

[55] Mahmoudian RA Akhlaghipour I Lotfi M Shahidsales S Moghbeli M . Circular RNAs as the pivotal regulators of epithelial-mesenchymal transition in gastrointestinal tumor cells. Pathol Res Pract. (2023) 245:154472. doi: 10.1016/j.prp.2023.154472 37087995

[56] Xu W Zhu L Zhang S Wang X Gong D Fan Y . Advances in the roles and molecular mechanisms of exosomal circular RNAs in regulating the pre-metastatic niche of tumors. Discov Oncol. (2025) 16:568. doi: 10.1007/s12672-025-02374-w 40252161 PMC12009264

[57] Mao Y Wang J Wang Y Fu Z Dong L Liu J . Hypoxia induced exosomal Circ-ZNF609 promotes pre-metastatic niche formation and cancer progression via miR-150-5p/VEGFA and HuR/ZO-1 axes in esophageal squamous cell carcinoma. Cell Death Discov. (2024) 10:133. doi: 10.1038/s41420-024-01905-8 38472174 PMC10933275

[58] Zhou J Song Q Li H Han Y Pu Y Li L . Targeting circ-0034880-enriched tumor extracellular vesicles to impede SPP1(high)CD206(+) pro-tumor macrophages mediated pre-metastatic niche formation in colorectal cancer liver metastasis. Mol Cancer. (2024) 23:168. doi: 10.1186/s12943-024-02086-9 39164758 PMC11334400

[59] He T Tao W Zhang J Xia TL Xu B Li LY . Extracellular vesicle-mediated transmission of circPDLIM5 promotes lymphatic metastasis in prostate cancer. J Exp Clin Cancer Res. (2025) 44:188. doi: 10.1186/s13046-025-03443-2 40611320 PMC12224790

[60] Sherafat NS Mardi A Absalan F Mehmandar-Oskuie A . Circular RNAs as central regulators of metabolic reprogramming and immune responses in the breast cancer microenvironment: A comprehensive review. BioMed Pharmacother. (2025) 193:118682. doi: 10.1016/j.biopha.2025.118682 41175594

[61] Gao X Sun Z Liu X Luo J Liang X Wang H . 127aa encoded by circSpdyA promotes FA synthesis and NK cell repression in breast cancers. Cell Death Differ. (2025) 32:416–33. doi: 10.1038/s41418-024-01396-1 39402211 PMC11894148

[62] Jian H Li X Lei X Tang S Yang X . Mechanisms and therapeutic implications of ncRNAs in regulating the PD-1/PD-L1 axis across cancers. Comb Chem High Throughput Screen. (2026) 29:1141–51. doi: 10.2174/0113862073384743250617062430 40598725

[63] Fu J Liu F Bai S Jiang X Song H Zhang M . Circular RNA CDYL facilitates hepatocellular carcinoma stemness and PD-L1(+) exosomes-mediated immunotherapy resistance via stabilizing hornerin protein by blocking synoviolin 1-mediated ubiquitination. Int J Biol Macromol. (2025) 310:143246. doi: 10.1016/j.ijbiomac.2025.143246 40250664

[64] Li L Zhang Q Lian K . Circular RNA circ_0000284 plays an oncogenic role in the progression of non-small cell lung cancer through the miR-377-3p-mediated PD-L1 promotion. Cancer Cell Int. (2020) 20:247. doi: 10.1186/s12935-020-01310-y 32550825 PMC7298744

[65] Wang C Ju C Du D Zhu P Yin J Jia J . CircNF1 modulates the progression and immune evasion of esophageal squamous cell carcinoma through dual regulation of PD-L1. Cell Mol Biol Lett. (2025) 30:37. doi: 10.1186/s11658-025-00712-y 40158127 PMC11955112

[66] Yu H Zhang Y Yang R Xie C Liao Z Zhou T . CircENTPD7 affects the immune escape of non‐small cell lung cancer cells by modulating the IGF2BP2/PD‐L1 axis. Oncol Lett. (2025) 29:112. doi: 10.3892/ol.2024.14858 39802311 PMC11718618

[67] Yu L Sun M Zhang Q Zhou Q Wang Y . Harnessing the immune system by targeting immune checkpoints: Providing new hope for Oncotherapy. Front Immunol. (2022) 13:982026. doi: 10.3389/fimmu.2022.982026 36159789 PMC9498063

[68] Liu Z Zheng N Li J Li C Zheng D Jiang X . N6-methyladenosine-modified circular RNA QSOX1 promotes colorectal cancer resistance to anti-CTLA-4 therapy through induction of intratumoral regulatory T cells. Drug Resist Update. (2022) 65:100886. doi: 10.1016/j.drup.2022.100886 36370665

[69] Wang J Zhao X Wang Y Ren F Sun D Yan Y . circRNA-002178 act as a ceRNA to promote PDL1/PD1 expression in lung adenocarcinoma. Cell Death Dis. (2020) 11:32. doi: 10.1038/s41419-020-2230-9 31949130 PMC6965119

[70] Hu Z Chen G Zhao Y Gao H Li L Yin Y . Exosome-derived circCCAR1 promotes CD8 + T-cell dysfunction and anti-PD1 resistance in hepatocellular carcinoma. Mol Cancer. (2023) 22:55. doi: 10.1186/s12943-023-01759-1 36932387 PMC10024440

[71] Zhang PF Gao C Huang XY Lu JC Guo XJ Shi GM . Cancer cell-derived exosomal circUHRF1 induces natural killer cell exhaustion and may cause resistance to anti-PD1 therapy in hepatocellular carcinoma. Mol Cancer. (2020) 19:110. doi: 10.1186/s12943-020-01222-5 32593303 PMC7320583

[72] Chen Q Mang G Wu J Sun P Li T Zhang H . Circular RNA circSnx5 controls immunogenicity of dendritic cells through the miR-544/SOCS1 axis and PU.1 activity regulation. Mol Ther. (2020) 28:2503–18. doi: 10.1016/j.ymthe.2020.07.001 32681834 PMC7646215

[73] Wei X Xiang X Wang H Wang Z Xing S Peng W . Tumor cell-intrinsic circular RNA circFNDC3B attenuates CD8(+) T cells infiltration in non-small cell lung cancer. Commun Biol. (2025) 8:711. doi: 10.1038/s42003-025-08108-6 40341878 PMC12062398

[74] Heidari-Foroozan M Rezalotfi A Rezaei N . The molecular landscape of T cell exhaustion in the tumor microenvironment and reinvigoration strategies. Int Rev Immunol. (2024) 43:419–40. doi: 10.1080/08830185.2024.2401352 39257319

[75] Jarnicki AG Lysaght J Todryk S Mills KH . Suppression of antitumor immunity by IL-10 and TGF-beta-producing T cells infiltrating the growing tumor: influence of tumor environment on the induction of CD4+ and CD8+ regulatory T cells. J Immunol. (2006) 177:896–904. doi: 10.4049/jimmunol.177.2.896 16818744

[76] Zhou L Liu M Liu F Wang Z Li X Peng X . Circular RNA CLASP1 modulates the GLI1/SNAIL axis and enhances macrophage polarization in breast cancer. Oncogene. (2025) 44:4765–80. doi: 10.1038/s41388-025-03627-2 41249488

[77] Huang M Huang X Huang N . Exosomal circGSE1 promotes immune escape of hepatocellular carcinoma by inducing the expansion of regulatory T cells. Cancer Sci. (2022) 113:1968–83. doi: 10.1111/cas.15365 35396771 PMC9207376

[78] Wei CY Zhu MX Lu NH Liu JQ Yang YW Zhang Y . Circular RNA circ_0020710 drives tumor progression and immune evasion by regulating the miR-370-3p/CXCL12 axis in melanoma. Mol Cancer. (2020) 19:84. doi: 10.1186/s12943-020-01191-9 32381016 PMC7204052

[79] Liu Y Zhang H Zhang W Xiang L Yin Z Xu H . circ_0004140 promotes LUAD tumor progression and immune resistance through circ_0004140/miR-1184/CCL22 axis. Cell Death Discov. (2022) 8:181. doi: 10.1038/s41420-022-00983-w 35396377 PMC8993797

[80] Lv C Chen J Wang Y Lin Y . Immunoregulatory role of exosomal circRNAs in the tumor microenvironment. Front Oncol. (2025) 15:1453786. doi: 10.3389/fonc.2025.1453786 40034598 PMC11872884

[81] Li J Zhou W Wang H Huang M Deng H . Exosomal circular RNAs in tumor microenvironment: An emphasis on signaling pathways and clinical opportunities. MedComm (2020). (2024) 5:e70019. doi: 10.1002/mco2.70019 39584047 PMC11586091

[82] Samad MA Ahmad I Suhail M Al-Abbasi FA Tabrez S . Role of circular RNAs in regulating tumor microenvironment, epithelial mesenchymal transition, and resistance to cancer therapy. Crit Rev Oncol Hematol. (2025) 216:104942. doi: 10.1016/j.critrevonc.2025.104942 40953760

[83] Liu S Wan B Xie C Dong F . Targeting the circC16orf62/miR-1299/PHF8 axis to suppress metastasis and overcome immune evasion in triple-negative breast cancer. BioMed Pharmacother. (2026) 199:119392. doi: 10.1016/j.biopha.2026.119392 42008953

[84] Wang X Yang X Xing L Chen H Xie F Wang B . CircLRBA promotes epithelial-mesenchymal transition, immune evasion, chemoimmunotherapy resistance and metastasis through stabilizing Twist1. Adv Sci (Weinh). (2025) 12:e08918. doi: 10.1002/advs.202508918 41082269 PMC12752550

[85] Zhang YC Zhang YT Wang Y Zhao Y He LJ . What role does PDL1 play in EMT changes in tumors and fibrosis? Front Immunol. (2023) 14:1226038. doi: 10.3389/fimmu.2023.1226038 37649487 PMC10463740

[86] Chen RQ Xu XH Liu F Li CY Li YJ Li XR . The binding of PD-L1 and Akt facilitates glioma cell invasion upon starvation via Akt/Autophagy/F-Actin signaling. Front Oncol. (2019) 9:1347. doi: 10.3389/fonc.2019.01347 31850228 PMC6901431

[87] Li J Dong X Kong X Wang Y Li Y Tong Y . Circular RNA hsa_circ_0067842 facilitates tumor metastasis and immune escape in breast cancer through HuR/CMTM6/PD-L1 axis. Biol Direct. (2023) 18:48. doi: 10.1186/s13062-023-00397-3 37592296 PMC10436663

[88] He Y Tang J . Abstract P5-07-12: Circular RNA NCAPG promotes breast cancer metastasis through acting as the sponge of miR-200s. Cancer Res. (2019) 79:P5-07-12-P05-07-12. doi: 10.1158/1538-7445.Sabcs18-p5-07-1236230740

[89] Sun Y Li Y Niu T Wang Z Yang J He Y . CircNSD2 promotes metastasis and immune escape by increasing the USP10/SRSF6-mediated alternative splicing of TPM1 in TNBC. Mol Cancer. (2026) 25:111. doi: 10.1186/s12943-026-02627-4 41808072 PMC13088798

[90] Wang D Wang S Jin M Zuo Y Wang J Niu Y . Hypoxic exosomal circPLEKHM1-mediated crosstalk between tumor cells and macrophages drives lung cancer metastasis. Adv Sci (Weinh). (2024) 11:e2309857. doi: 10.1002/advs.202309857 38509870 PMC11165461

[91] Huang X Wang J Guan J Zheng Z Hao J Sheng Z . Exosomal Circsafb2 reshaping tumor environment to promote renal cell carcinoma progression by mediating M2 macrophage polarization. Front Oncol. (2022) 12:808888. doi: 10.3389/fonc.2022.808888 35646637 PMC9133324

[92] Luo X Peng Y Fan X Xie X Jin Z Zhang X . The crosstalk and clinical implications of circRNAs and glucose metabolism in gastrointestinal cancers. Cancers (Basel). (2023) 15. doi: 10.3390/cancers15082229 37190158 PMC10136712

[93] Shen H Liu B He J Zhao W Li W Ma L . Lactate metabolic reprogramming mediated by circRNA-LDHA complex facilitates innate immune evasion of liver cancer. Adv Sci (Weinh). (2025) 12:e09989. doi: 10.1002/advs.202509989 40940312 PMC12677679

[94] Li H Guo H Huang Q Wang S Li X Qiu M . Circular RNA P4HB promotes glycolysis and tumor progression by binding with PKM2 in lung adenocarcinoma. Respir Res. (2023) 24:252. doi: 10.1186/s12931-023-02563-7 37880717 PMC10601333

[95] Li J Xu S Zhan Y Lv X Sun Z Man L . CircRUNX1 enhances the Warburg effect and immune evasion in non-small cell lung cancer through the miR-145/HK2 pathway. Cancer Lett. (2025) 620:217639. doi: 10.1016/j.canlet.2025.217639 40090573

[96] Lan T Gao F Cai Y Lv Y Zhu J Liu H . The protein circPETH-147aa regulates metabolic reprogramming in hepatocellular carcinoma cells to remodel immunosuppressive microenvironment. Nat Commun. (2025) 16:333. doi: 10.1038/s41467-024-55577-0 39747873 PMC11696079

[97] Dodbele S Mutlu N Wilusz JE . Best practices to ensure robust investigation of circular RNAs: pitfalls and tips. EMBO Rep. (2021) 22:e52072. doi: 10.15252/embr.202052072 33629517 PMC7926241

[98] Chuang TJ Chiang TW Chen CY . Assessing the impacts of various factors on circular RNA reliability. Life Sci Alliance. (2023) 6. doi: 10.26508/lsa.202201793 36849251 PMC9971162

[99] Zhang Y Nguyen TM Zhang XO Wang L Phan T Clohessy JG . Optimized RNA-targeting CRISPR/Cas13d technology outperforms shRNA in identifying functional circRNAs. Genome Biol. (2021) 22:41. doi: 10.1186/s13059-021-02263-9 33478577 PMC7818937

[100] Seyhan AA . Circulating liquid biopsy biomarkers in glioblastoma: advances and challenges. Int J Mol Sci. (2024) 25. doi: 10.3390/ijms25147974 39063215 PMC11277426

[101] Dong Y Gao Q Chen Y Zhang Z Du Y Liu Y . Identification of circRNA signature associated with tumor immune infiltration to predict therapeutic efficacy of immunotherapy. Nat Commun. (2023) 14:2540. doi: 10.1038/s41467-023-38232-y 37137884 PMC10156742

[102] Li J Zhang G Liu CG Xiang X Le MTN Sethi G . The potential role of exosomal circRNAs in the tumor microenvironment: insights into cancer diagnosis and therapy. Theranostics. (2022) 12:87–104. doi: 10.7150/thno.64096 34987636 PMC8690929

[103] Chang Y Ding J . CircRNA knockout/knockdown tools in molecular biology research. Biochem Biophys Res Commun. (2025) 783:152607. doi: 10.1016/j.bbrc.2025.152607 40929845

[104] Han R Rao X Zhou H Lu L . Synergistic immunoregulation: harnessing circRNAs and piRNAs to amplify PD-1/PD-L1 inhibition therapy. Int J Nanomedicine. (2024) 19:4803–34. doi: 10.2147/ijn.S461289 38828205 PMC11144010

[105] Wang F Lu Q Yu H Zhang XM . The circular RNA circFGFR4 facilitates resistance to anti-PD-1 of triple-negative breast cancer by targeting the miR-185-5p/CXCR4 axis. Cancer Manag Res. (2023) 15:825–35. doi: 10.2147/cmar.S411901 37601820 PMC10439764

[106] Huang D Zhu X Ye S Zhang J Liao J Zhang N . Tumour circular RNAs elicit anti-tumour immunity by encoding cryptic peptides. Nature. (2024) 625:593–602. doi: 10.1038/s41586-023-06834-7 38093017

[107] Welsh JA Goberdhan DCI O'Driscoll L Buzas EI Blenkiron C Bussolati B . Minimal information for studies of extracellular vesicles (MISEV2023): From basic to advanced approaches. J Extracell Vesicles. (2024) 13:e12404. doi: 10.1002/jev2.12404 38326288 PMC10850029

[108] Bustin SA Ruijter JM van den Hoff MJB Kubista M Pfaffl MW Shipley GL . MIQE 2.0: Revision of the minimum information for publication of quantitative real-time PCR experiments guidelines. Clin Chem. (2025) 71:634–51. doi: 10.1093/clinchem/hvaf043 40272429

[109] Pfaffl MW . How laboratory guidelines promote the validity of circulating extracellular vesicle-associated nucleic acid biomarker signatures in liquid biopsy. Int J Mol Sci. (2025) 26. doi: 10.3390/ijms262412115 41465539 PMC12733191

[110] Niu D Wu Y Lian J . Circular RNA vaccine in disease prevention and treatment. Signal Transduct Target Ther. (2023) 8:341. doi: 10.1038/s41392-023-01561-x 37691066 PMC10493228

